# Synthesis and Pharmacological Activity of Diterpenylnaphthoquinone Derivatives

**DOI:** 10.3390/molecules16108614

**Published:** 2011-10-13

**Authors:** Mariano Walter Pertino, Cristina Theoduloz, Jose Antonio Palenzuela, Maria del Mar Afonso, Erdem Yesilada, Francisco Monsalve, Paulo González, Daniel Droguett, Guillermo Schmeda-Hirschmann

**Affiliations:** 1 Instituto de Química de Recursos Naturales, Universidad de Talca, Casilla 747, Talca, Chile; 2 Facultad de Ciencias de la Salud, Universidad de Talca, Casilla 747, Talca, Chile; 3 Instituto Universitario de Bio-Orgánica Antonio Gonzalez G., Universidad de La Laguna, Tenerife 38206, Spain; 4 Faculty of Pharmacy, Yeditepe University, Istanbul 34755, Turkey

**Keywords:** labdane diterpenes, lapachol derivatives, diterpenylnaphthoquinones, gastroprotection, basal cytotoxicity

## Abstract

New diterpenylquinones, combining a diterpene diacid and a naphthoquinone, were prepared from junicedric acid and lapachol. The new derivatives were assessed as gastroprotective agents by the HCl-EtOH-induced gastric lesions model in mice as well as for basal cytotoxicity on the following human cell lines: Normal lung fibroblasts (MRC-5), gastric epithelial adenocarcinoma (AGS), and hepatocellular carcinoma (Hep G2). Several of the new compounds were significantly active as antiulcer agents and showed selective cytotoxicity against AGS cells.

## 1. Introduction

In the year 2002 stomach cancer and peptic ulcer disease represented 1.5 and 0.5% of the total causes of death worldwide, respectively [1]. Chronic gastric ulcers can lead to gastric and pancreatic cancer [2,3]. Several natural products have been shown to display significant gastroprotective effects in animal models of induced gastric ulcers. These agents include diterpenes with different structural skeletons [4,5,6,7,8,9,10,11]. The labdane diterpenes from *Araucaria araucana* (Molina) Koch (Araucariaceae) and their semisynthetic derivatives present gastroprotective activity with a wide range of basal cytotoxicity on human cell lines [9,10]. According to Halle and Spielmann there is a significant correlation between cytotoxicity of mammalian cell culture systems and acute oral toxicity (LD_50_) in animals [12]. Basal cytotoxicity is a valid model to predict starting doses for *in vivo* lethality assays in rodents.

Lapachol is a naphthoquinone that can be obtained in high yields from the wood or trunk bark of *Tabebuia* species (Bignoniaceae), including *Tabebuia heptaphylla* (Vell. Conc.) Toledo [13]. Several bioactivities have been described for lapachol and its semisynthetic derivatives, e.g., activation of the Epstein-Barr virus [14], molluscicidal [15,16], trypanocidal [17], antiviral, antiproliferative [18], and DNA-topoisomerase inhibitory activity [19].

A new approach to drug design is to link two molecules with individual intrinsic effect into a single compound, named a “hybrid compound” [20]. A classification of “hybrid” molecules includes conjugates, cleavage conjugates, fused hybrid molecules, and merged hybrids [20,21,22,23]. The use of lapachol to obtain lapachol hybrid derivatives with diterpenes has not been explored previously, but changes in the gastroprotective effect and basal cytotoxicity of the resulting products should be expected.

In this paper we describe the preparation of junicedric acid and lapachol derivatives and examine the structure-activity relationships/trends of the new compounds as gastroprotective agents in mice. The basal cytotoxicity of the new compounds was also assessed towards the following human cell lines: normal lung fibroblasts (MRC-5), epithelial adenocarcinoma (AGS), and hepatocellular carcinoma (Hep G2).

## 2. Results and Discussion

**Scheme 1:**
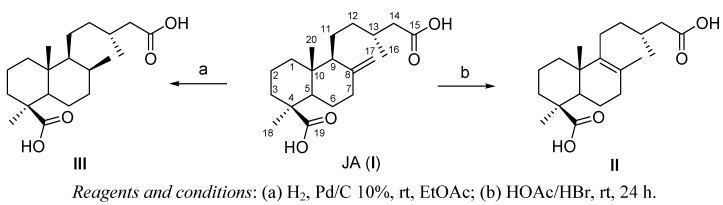
Preparation of derivatives **II** and **III** from junicedric acid (**I**).

**Scheme 2:**
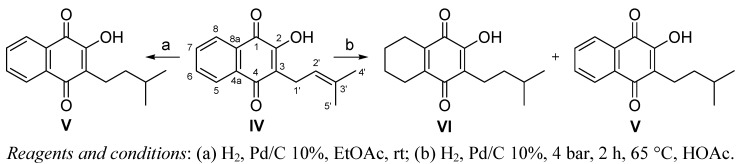
Preparation of derivatives **V** and **VI** from lapachol (**IV**).

The diterpene junicedric acid (**I**) (Scheme 1) was obtained by saponification and oxidation of a mixture of labdane diterpenes from *Araucaria araucana* [9,10]. Isomerization of the double bond of **I** to **II** was carried out by treating **I** in acetic acid (HOAc) with HBr. Reduction of the double bond of **I** was carried out by catalytic hydrogenation of the terpene (Scheme 1). Lapachol (2-hydroxy-3-(3-methyl-2-butenyl)-[1,4]-naphthoquinone) (**IV**, Scheme 2) was obtained from “lapacho” wood extract [13]. The hydrogenated products were obtained treating lapachol in ethyl acetate (EtOAc) with palladium on activated carbon (Pd/C) (Scheme 2).

**Figure 1:**
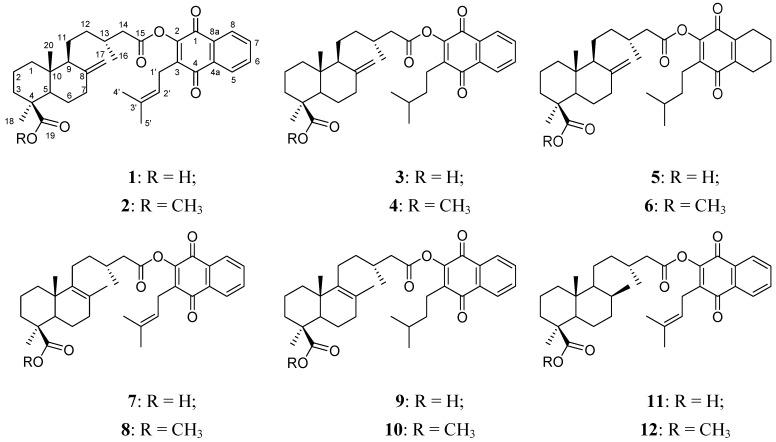
Structures of compounds **1–12**.

Twelve esters combining a diterpene and lapachol or its derivatives were prepared in moderate to good yields starting from the diterpene diacids **I**, **II** or **III**. The quinone moieties used included 2-hydroxy-3-(3-methyl-2-butenyl)-[1,4]-naphthoquinone (lapachol) (**IV**), 2-hydroxy-3-(3-methyl-butyl)-[1,4]-naphthoquinone (**V**) (dihydroprenyllapachol) and 2-hydroxy-3-(3-methyl-butyl)-5,6,7,8-tetrahydro-[1,4]-naphthoquinone (**VI**) (dihydroprenyl-5,6,7,8-tetrahydrolapachol) (Scheme 2). All the products were characterized by spectroscopic means. Compounds **1–12** (Figure 1) are described for the first time.

The diterpene junicedric acid has two acid groups at C-15 and C-19. However, only the acid group at C-15 is reactive enough to form esters or amides when the diacid is treated with *N*,*N*-dicyclohexyl-carbodiimide (DCC)/dimethylaminopyridine (DMAP) and the alcohols or amines. Diesters or diamides can be obtained via the acyl chloride [24].

Compounds **1–12** were evaluated for gastroprotective effect and basal cytotoxicity (Table 1 and Table 2).

When assayed for gastroprotective effect, lapachol (**IV**) at 5, 10 and 50 mg/kg did not present a statistically significant effect in the EtOH-HCl-induced gastric lesions model in mice when compared with the vehicle control (Tween 80). Comparisons were made using Dunnett’s or Student-Newman-Keuls test (data not shown). Compound **II** reduced gastric lesions by 79% at 5 mg/kg. The diterpene-lapachol hybrid derivatives were investigated for gastroprotective effect at a single oral dose of 5 mg/kg (Table 1).

**Table 1:** Gastroprotective effect of compounds **1–12** on the HCl-EtOH-induced gastric lesion model in mice. All compounds were assessed at a single oral dose of 5 mg/kg.

Compound	Lesion index (mm) Mean ± SEM	Protection (%)	Protected stomachs ^a^
Tween	27.2 ± 9.3		
Δ8(9) junicedric acid (**II**)	5.3 ± 2.4 *	79	4/9
**1**	15.3 ± 5.0	44	
Tween	25.0 ± 6.9		1/14
**2**	8.7 ± 3.9	65	4/9
**5**	20.4 ± 3.6	18	0/9
**6**	6.5 ± 2.7 *	74	3/8
**7**	11.5 ± 4.4	54	1/8
**8**	3.9 ± 1.9 *	84	5/9
**11**	23.5 ± 3.6	6	0/10
**12**	27.4 ± 6.8	-	0/7
Tween	43.3 ± 8.2		0/9
**3**	17.9 ± 4.6 **	59	0/8
**4**	19.3 ± 5.0 **	55	0/9
**9**	15.7 ± 4.5 **	64	0/9
**10**	10.2 ± 3.5 **	76	3/9
Lansoprazole (20 mg/kg)	9.4 ± 1.2 **	73	6/9

* and **: *P* < 0.05 different from untreated control (Tween); ^a^ Number of stomachs which were completely protected from any visible bleeding. One-way ANOVA with Student-Newman-Keuls *post-hoc* test.

Derivatives **6** and **8** were significantly active, protecting 3/8 and 5/9 animals, respectively, from lesions or stomach bleeding. An important gastroprotective effect was seen with derivatives **3**, **4**, **9** and **10** compared to untreated controls. However, for compounds **3**, **4** and **9** all the stomachs showed at least some lesions or bleeding. Compound **10** was a very active gastroprotective agent. Compounds **8** and **10** are methyl esters of **II** and **IV**, the quinone moiety bearing either the prenyl side chain or the reduced prenyl side chain. Compound **6** was built from **I** and **VI**. It showed very low basal cytotoxicity (>1 mM) and proved to be one of the best gastroprotective products found in this study. The most active compounds are C19 methyl esters of the parent compounds. This fact shows that changes in the structure of any of the moieties will lead to changes in activity of the new compounds.

The diterpenylnaphthoquinones as well as **I**, **II** and **IV** were evaluated for basal cytotoxicity using MRC-5 fibroblasts, AGS and Hep G2 cells (Table 2). No significant differences in basal cytotoxicity were found between derivatives **1** and **7**, where the diterpene moiety presents the double bond either at 8(17)-en- (compound **1**) or at 8(9)-en- (compound **7**).

**Table 2:** Basal cytotoxicity of the lapachoyl ester derivatives from labdane diterpenes **1–12** towards MRC-5 fibroblasts and AGS and Hep G2 cells.

Compound	IC_50_ ± SEM ^a^ (µM)		
	Fibroblasts	AGS	Hep G2
Lapachol (**IV**)	>1000	382 ± 15	55 ± 3
Junicedric acid (**I**)	181 ± 9	304 ± 18	>1000
Δ8(9) junicedric acid (**II**)	214 ± 32	343 ± 22	>1000
**1**	210 ± 13	170 ± 9	57.4 ± 4.4
**2**	741 ± 40	361 ± 19	208 ± 11
**3**	156 ± 9	89 ± 5	>1000
**4**	>1000	382 ± 26	>1000
**5**	69 ± 3	40 ± 6	27 ± 1
**6**	>1000	>1000	>1000
**7**	190 ± 8	179 ± 9	96 ± 9
**8**	>1000	721 ± 35	379 ± 21
**9**	341 ± 17	294 ± 15	>1000
**10**	>1000	162 ± 10	>1000
**11**	336 ± 15	114 ± 7	54.5 ± 5
**12**	926 ± 35	323 ± 16	290 ± 9
Etoposide	0.33 ± 0.02	0.58 ± 0.02	-
Lansoprazole ^b^	306 ± 11	162 ± 6	221 ± 9

^a^ Confluent cultures were treated with the culture medium containing the compounds at concentrations ranging between 0 and 1,000 µM for 24 h. Cell viability was determined by the neutral red uptake assay. Data are expressed as arithmetic mean values of three different experiments in quadruplicate ± SEM; ^b^ Reference compound.

Cytotoxicity strongly decreases after methylation, showing the relevance of the COOH function at C-19 for this effect. In a broad sense, all compounds displayed lower basal cytotoxicity against fibroblasts than against AGS or Hep G2 cells. Some selectivity was seen for compounds **1**, **5**, **7** and **11** towards Hep G2 cells. The most cytotoxic compound of this series (**5**) was built with **I** and the hydrogenation product of lapachol **VI**. The IC_50_ values of this compound towards fibroblasts, AGS and Hep G2 cells were 69, 40 and 27 µM, respectively. The increase in cytotoxicity was associated with poor selectivity. The effect is completely lost after methylation of the C-19 COOH function of the diterpene.

A similar approach, but starting from myrcecommunic acid and *p*-benzoquinone was used to prepare new diterpenylnaphthoquinones through a Diels-Alder cycloaddition [25]. Several of the diterpenylnaphthoquinones presented higher cytotoxicity than the quinone moiety itself, the naphthoquinone (NQ) system showing better effect than benzoquinone or anthraquinone derivatives [25,26]. The most promising compounds present variations in the decaline part of the naphthohydroquinone (NHQ) of the NQ system and show GI_50_ values (growth inhibition values that reduce cell growth by 50%) in the 0.12–0.50 µM range towards the tumour cell lines A-549 (lung carcinoma), HT-29 (colon carcinoma), and MEL-28 (malignant melanoma) [25]. The main difference with this report is that [25] formed C-C bonds by cycloaddition, while we prepared esters of dicarboxylic diterpenes and napththoquinones.

## 3. Experimental

### 3.1. General Experimental Procedures

Optical rotations were obtained for solutions in CHCl_3_ (concentrations expressed in g/100 mL) on a Jasco DIP 370 polarimeter (Jasco Analytical Instruments, Easton, MD, USA). IR spectra were recorded on a Nicolet Nexus FT-IR instrument (Thermo Electron Corporation, Whaltham, MA, USA). ^1^H-NMR spectra were recorded at 400 or 500 MHz and ^13^C-NMR data were obtained at 100 or 125 MHz on a Bruker Avance spectrometer (Bruker, Rheinstetten, Germany). Chemical shifts are given in δ (ppm) with TMS as the internal standard. Mass spectra were measured with an EBE trisector VG Autospec Micromass spectrometer operating at 70 eV and are presented as *m/z* (rel. int. %). Silica gel 60 (Merck, 63–200 µm particle size) was used for column chromatography, precoated silica gel plates (Merck, Kieselgel 60 F_254_, 0.25 mm) were used for thin layer chromatography (TLC). TLC spots were visualized by spraying the chromatograms with *p*-anisaldehyde-ethanol-acetic acid-H_2_SO_4_ (2:170:20:10 v/v) and heating at 110 °C for 3 min. 1,3-Dicyclohexylcarbodiimide (DCC) and dimethylaminopyridine (DMAP) were from Merck (Schuchardt, Germany).

### 3.2. Plant Material

Lapachol was isolated from the wood of *Tabebuia heptaphylla* as described previously [13] and purified by successive silica gel column chromatography, followed by crystallization. The diterpene junicedric acid (**I**) was obtained by saponification and oxidation of a mixture of labdane diterpenes from *Araucaria araucana* resin. The resin was collected from healthy trees in Conguillío, Araucanía Region, Chile. Voucher specimens have been deposited at the Herbarium of the Universidad de Talca.

### 3.3. General Procedure for the Synthesis of Compounds ***I–III*** and ***V–VI***

The crude *Araucaria araucana* resin was worked-up as described previously [9,10] to obtain a mixture of diterpenes bearing an alcohol, aldehyde, acid or ester (acetate) function at C-15 and/or C-19. After saponification (KOH, methanol), the diterpene mixture was oxidized with CrO_3_ to yield **I**. Isomerization of the double bond of **I** to **II** was carried out by treating **I** in acetic acid with 47% HBr, stirring constantly for 24 h. The reaction product was purified by silica gel column chromatography (86% w/w yield). Reduction of the double bond of **I** and **IV** was carried out by catalytic hydrogenation of the terpene dissolved in ethyl acetate with 10% Pd/C in a 1:10 molar ratio with respect to the diterpene, stirring constantly for 24 h to yield **III** and **V**, respectively. Compound **VI** was obtained by high pressure hydrogenation of compound **IV**.

*Δ8(9) Junicedric acid* (**II**). HBr (3 mL) was added to a solution of junicedric acid (**I**) (2.51 g, 7.47 mmol) in acetic acid (HOAc, 20 mL). The mixture was stirred at room temperature for 24 h, cooled in an ice bath, and after addition of water the aqueous phase was extracted with EtOAc (3 × 20 mL). The extract was dried over anhydrous Na_2_SO_4_, and taken to dryness under reduced pressure. The residue was purified by silica gel column chromatography, eluting with hexane/EtOAc (8:2), yielding 2.15 g (86%) of compound **II**.

*17β-Dihydrojunicedric acid* (**III**). Junicedric acid (**I**) (1.10 g, 3.27 mmol) was dissolved in 30 mL ethyl acetate. After the addition of Pd/C (10%) vacuum was made to eliminate air. Then, hydrogen (H_2_) was bubbled under constant agitation for 24 h. The reaction mixture was filtered and taken to dryness under reduced pressure. The residue was purified by silica gel column chromatography eluting with hexane/EtOAc (8:2), yielding **III** (827 mg, 75%).

*2-Hydroxy-3-(3-methylbutyl)-[1,4]-naphthoquinone* (**V**). Compound **V** (dihydroprenyl lapachol) was synthesized from 2-hydroxy-3-(3-methyl-2-butenyl)-[1,4]-naphthoquinone (lapachol) (**IV**) as described for compound **III**, yielding 306 mg (70%) of **V**.

*2-Hydroxy-3-(3-methyl-butyl)-5,6,7,8-tetrahydro)-[1,4]-naphthoquinone* (**VI**). A solution of lapachol (**IV**) (400 mg, 1.65 mmol) in HOAc (4 mL) and Pd/C (10%) was submitted to a pressure of 4 bar and 65 °C for 2 h. The reaction mixture was cooled in an ice-bath and after addition of water, the aqueous phase was extracted with EtOAc (3 × 20 mL), the extracts were dried over anhydrous Na_2_SO_4_, and taken to dryness under reduced pressure. The residue was purified by silica gel column chromatography eluting with hexane/EtOAc (9:1), yielding dihydroprenyl-5,6,7,8-tetrahydrolapachol **VI** (300 mg, 73%) and **V** (40 mg, 10%).

### 3.4. General Procedure for the Synthesis of Compounds ***1–12***

Compounds **1**, **3**, **5**, **7**, **9** and **11** were prepared by treating the corresponding diacids (1 mEq) in dry CH_2_Cl_2_ (DCM) with 1,3-dicyclohexylcarbodiimide (DCC) (1 mEq) at room temperature under constant stirring. After 10 min, lapachol or its reduction products (1 mEq) dissolved in dry DCM, were added together with a catalytic amount of dimethylaminopyridine (DMAP). After 2–4 h, the reaction was stopped by adding water and extracted with DCM. The extract was dried over Na_2_SO_4_, and purified by silica gel column chromatography, to afford compounds **1**, **3**, **5**, **7**, **9** and **11** in 33, 42, 35, 48, 52 and 42% w/w yields, respectively. The corresponding methyl esters **2**, **4**, **6**, **8**, **10** and **12** were obtained treating the above cited compounds with diazomethane in 92, 89, 88, 93, 88 and 90% w/w yields, respectively. The purity of all the derivatives was over 95%, as determined by ^1^H-NMR spectroscopy.

*Lapachoyl junicedrate* (**1**). Junicedric acid (**I**) (170 mg, 0.506 mmol), DCC (104 mg, 0.506 mmol), a catalytic amount of DMAP and lapachol (**IV**) (120 mg, 0.506 mmol), were stirred at room temperature in dry CH_2_Cl_2_ (20 mL) for 2–4 h. The reaction mixture was cooled in an ice bath. After addition of water, the aqueous phase was extracted with EtOAc (3 × 20 mL). The extract was dried over anhydrous Na_2_SO_4_ and taken to dryness under reduced pressure. The residue was purified by silica gel column chromatography, eluting with hexane/EtOAc (8:2), yielding **1** (94 mg, 33%): brown oil; [α]^20^_D_ +22 (*c* 0.24, CHCl_3_); IR ν_max_ (film) 3398, 2928, 2840, 1768, 1692, 1672, 1641, 1445, 1286, 1170, 1075 cm^−1^; ^1^H-NMR (CDCl_3_): see Table 3; ^13^C-NMR (CDCl_3_): 39.10 (C-1), 19.90 (C-2), 37.88 (C-3), 44.18 (C-4), 56.27 (C-5), 26.02 (C-6), 38.72 (C-7), 148.01 (C-8), 56.33 (C-9), 40.88 (C-10), 21.14 (C-11), 35.86 (C-12), 31.00 (C-13), 40.58 (C-14), 170.42 (C-15), 19.96 (C-16), 106.59 (C-17), 29.07 (C-18), 178.45 (C-19), 12.79 (C-20), Quinone: 184.39, 184.52 (C-1 and C-4), 150.87 (C-2), 138.31 (C-3), 130.84, 132.00 (C-4a, C-8a), 126.73, 126.59 (C-5 and C-8), 134.88, 134.13 (C-6 and C-7), 23.67 (C-1′), 118.45 (C-2′), 133.86 (C-3′), 18.05 (C-4′), 25.85 (C-5′); EIMS *m/z* 560 [M]^+^ (5), 290 (10), 244 (24), 243 (21), 242 (100), 228 (14), 227 (85), 225 (14), 123 (10), 121 (27), 109 (13), 105 (13), 95 (10), 81 (15), 55 (13); HREIMS *m/z* 560.3168 (calcd for C_35_H_44_O_6_, 560.3138).

**Table 3:** Selected ^1^H-NMR data of compounds **1–6**.

H	1	2	3	4	5	6
13	2.10 m	2.11 m	2.10 m	2.08 m	2.05 m	2.06 m
14 α	2.73 dd	2.73 dd	2.71 dd	2.71 dd	2.65 dd	2.66 dd
14 β	2.41-2.46 m	2.42-2.47 m	2.52 m	2.52 m		
16	1.13 d (6.6)	1.13 d (6.6)	1.10 d (6.6)	1.10 d (6.6)	1.07 d (6.6)	1.08 d (6.6)
17	4.86 s; 4.54 s	4.87 s; 4.54 s	4.85 s; 4.52 s	4.84 s; 4.51 s	4.84 s; 4.51 s	4.86 s; 4.51 s
18	1.26 s	1.20 s	1.23 s	1.17 s	1.24 s	1.20 s
20	0.63 s	0.54 s	0.61 s	0.51 s	0.60 s	0.59 s
OMe	-	3.64 s	-	3.61 s	-	3.63 s
Quinone						
5 and 8	8.10 m	8.10 m	8.08 m	8.08 m	2.42 m	2.44 m
6 and 7	7.74 m	7.74 m	7.71 m	7.71 m	1.69 m	1.70 m
1’	3.27 br d	3.28 br d	2.41 m	2.41 m	2.40 m	2.38 m
2’	5.07 br t	5.09 br t	1.34 m	1.36 m	1.33 m	1.32 m
3’	-	-	1.59 m	1.58 m	1.57 m	1.57 m
4’	1.77 s	1.77 s	0.93 d (6.6)	0.93 d (6.6)	0.91 d (6.6)	0.92 d (6.6)
5’	1.69 s	1.69 s	0.93 d (6.6)	0.93 d (6.6)	0.91 d (6.6)	0.92 d (6.6)

*J* (Hz): 14*α*, 14*β* = 15.3; 13, 14*α* = 5.5; 13, 14*β* = 8.1.

*Lapachoyl junicedrate methyl ester* (**2**). Compound **1** (50 mg, 0.089 mmol), was methylated with a solution of CH_2_N_2_ in ethyl ether, yielding 47 mg (92%) of **2**: brown oil; [α]^20^_D_ +24 (*c* 0.29, CHCl_3_); IR ν_max_ (film) 2948, 2844, 1766, 1720, 1676, 1641, 1449, 1290, 1178, 1079 cm^−1^; ^1^H-NMR (CDCl_3_): see Table 3; ^13^C-NMR (CDCl_3_): 39.21 (C-1), 19.94 (C-2), 38.28 (C-3), 44.33 (C-4), 56.38 (C-5), 26.28 (C-6), 38.79 (C-7), 148.11 (C-8), 56.45 (C-9), 40.87 (C-10), 21.15 (C-11), 35.90 (C-12), 30.98 (C-13), 40.39 (C-14), 170.24 (C-15), 19.99 (C-16), 106.46 (C-17), 28.84 (C-18), 177.77 (C-19), 12.59 (C-20), 51.14 (OMe), Quinone: 178.37, 184.44 (C-1 and C-4) 150.95 (C-2), 138.28 (C-3), 130.93, 132.09 (C-4a, C-8a), 126.68, 126.53 (C-5 and C-8), 134.73, 134.00 (C-6 and C-7), 23.65 (C-1′), 118.53 (C-2′), 133.74 (C-3′), 17.97 (C-4′), 25.73(C-5′); EIMS *m/z* 574 [M]^+^ (2), 350 (6), 291 (17), 290 (30), 258 (16), 256 (11), 243 (10), 242 (67), 227 (24), 200 (10), 180 (11), 161 (13), 159 (10), 123 (14), 122 (15), 121 (100), 109 (23), 107 (21), 105 (20), 95 (16), 93 (16), 91 (11), 81 (20), 79 (12), 67 (12), 55 (18); HREIMS *m/z* 574.3308 (calcd for C_36_H_46_O_6_, 574.3294).

*Dihydroprenyl lapachoyl junicedrate* (**3**). Compound **3** was synthesized as described for compound **1**, using terpene **I** and quinone **V**, yielding 105 mg (42%) of **3**: pale yellow oil; [α]^20^_D_ +49 (*c* 0.14, CHCl_3_); IR ν_max_ (film) 2952, 2872, 1772, 1700, 1680, 1641, 1469, 1290, 1186, 1079 cm^−1^; ^1^H-NMR (CDCl_3_): see Table 3;^13^C-NMR (CDCl_3_): 39.18 (C-1), 19.97 (C-2), 37.97 (C-3), 44.24 (C-4), 56.40 (C-5), 26.07 (C-6), 38.77 (C-7), 148.01 (C-8), 56.46 (C-9), 40.61 (C-10), 21.19 (C-11), 35.93 (C-12), 31.02 (C-13), 40.89 (C-14), 170. 36 (C-15), 19.96 (C-16), 106.60 (C-17), 29.06 (C-18), 178.16 (C-19), 12.79 (C-20), Quinone: 184.10, 184.57 (C-1 and C-4) 151.14 (C-2), 140.17 (C-3), 130.96, 132.16 (C-4a, C-8a), 126.68, 126.57 (C-5 and C-8), 134.01 133.78 (C-6 and C-7), 22.47 (C-1′), 37.51 (C-2′), 28.41 (C-3′), 22.35 (C-4′), 22.35 (C-5′); HREIMS *m/z*: 533.0547 [M-H-CO]^+^ (calcd for C_34_H_45_O_5_: 533.3267).

*Dihydroprenyl lapachoyl junicedrate methyl ester* (**4**). Compound **3** (60 mg, 0.106 mmol) was methylated with a solution of CH_2_N_2_ in ethyl ether, yielding 55 mg (89%) of **4**: pale yellow oil; [α]^20^_D_ +62 (*c* 0.22, CHCl_3_); IR ν_max_ (film) 2928, 2872, 1768, 1724, 1676, 1637, 1461, 1298, 1146, 1,083 cm^−1^; ^1^H-NMR (CDCl_3_): see Table 3; ^13^C-NMR (CDCl_3_): 39.23 (C-1), 19.97 (C-2), 37.51 (C-3), 44.35 (C-4), 56.40 (C-5), 26.29 (C-6), 38.81 (C-7), 148.11 (C-8), 56.46 (C-9), 40.41 (C-10), 21.19 (C-11), 35.93 (C-12), 31.02 (C-13), 40.89 (C-14), 170. 36 (C-15), 20.01 (C-16), 106.50 (C-17), 28.87 (C-18), 178.16 (C-19), 12.61 (C-20), 51.17 (OMe), Quinone: 184.10, 184.57 (C-1 and C-4) 151.11 (C-2), 140.17 (C-3), 130.96, 132.16 (C-4a, C-8a), 126.68, 126.56 (C-5 and C-8), 134.02, 133.78 (C-6 and C-7), 22.47 (C-1′), 37.51 (C-2′), 28.41 (C-3′), 22.35 (C-4′), 22.35 (C-5′); HREIMS *m/z*: 577.1957 [M+H]^+^ (calc for C_36_H_49_O_6_, 577.3529).

*Dihydroprenyl-5,6,7,8-tetrahydrolapachoyl junicedrate* (**5**). Compound **5** was synthesized as described for compound **1**, using terpene I and quinone **V**I, yielding 87 mg (35%) of **5**: pale yellow oil; [α]^20^_D_ +35 (*c* 0.55, CHCl_3_); IR ν_max_ (film) 3338, 2924, 2860, 1768, 1692, 1661, 1621, 1473, 1206, 1134, 1083 cm^−1^; ^1^H-NMR (CDCl_3_): see Table 3; ^13^C-NMR (CDCl_3_): 39.10 (C-1), 19.88 (C-2), 37.89 (C-3), 44.17 (C-4), 56.33 (C-5), 26.00 (C-6), 38.71 (C-7), 147.93 (C-8), 56.38 (C-9), 40.54 (C-10), 21.03 (C-11), 35.84 (C-12), 30.94 (C-13), 40.77 (C-14), 170.46 (C-15), 19.88 (C-16), 106.53 (C-17), 28.99 (C-18), 180.25 (C-19), 12.70 (C-20), Quinone: 184.24, 186.91 (C-1 and C-4) 148.60 (C-2), 140.85 (C-3), 136.89, 142.76 (C-4a, C-8a), 22.30, 21.03 (C-5 and C-8), 21.85, 21.11 (C-6 and C-7), 22.79 (C-1′), 37.46 (C-2′), 28.23 (C-3′), 22.26 (C-4′), 22.26 (C-5′); EIMS *m/z* 566 [M]^+^ (2), 273 (12), 251 (17), 250 (100), 248 (15), 193 (11), 192 (21), 121 (12). HREIMS *m/z* 566.3633 (calcd for C_35_H_50_O_6_, 566.3607).

*Dihydroprenyl-5,6,7,8-tetrahydrolapachoyl junicedrate methyl ester* (**6**). Compound **5** (40 mg, 0.071 mmol) was methylated with a solution of CH_2_N_2_ in ethyl ether, yielding 36 mg (88%) of **6**: pale yellow oil, [α]^20^_D_ +28 (*c* 0.20, CHCl_3_); IR ν_max_ (film) 2948, 2872, 1764, 1720, 1661, 1621, 1457, 1214, 1134, 1083 cm^−1^; ^1^H-NMR (CDCl_3_): see Table 3; ^13^C-NMR (CDCl_3_): 39.43 (C-1), 20.12 (C-2), 38.50 (C-3), 44.55 (C-4), 56.61 (C-5), 26.49 (C-6), 39.01 (C-7), 148.32 (C-8), 56.66 (C-9), 40.60 (C-10), 22.11 (C-11), 36.13 (C-12), 31.20 (C-13), 41.04 (C-14), 170.69 (C-15), 20.21 (C-16), 106.66 (C-17), 29.06 (C-18), 177.98 (C-19), 12.79 (C-20), 51.34 (OMe), Quinone: 180.52, 187.19 (C-1 and C-4) 148.86 (C-2), 141.11 (C-3), 137.15, 143.02 (C-4a, C- 8a), 23.04, 22.49 (C-5 and C-8), 21.28, 21.28 (C-6 and C-7), 21.37 (C-1′), 37.71 (C-2′), 28.49 (C-3′), 22.48 (C-4′), 22.48 (C-5′); EIMS *m/z* 580 [M]^+^ (2), 333 (13), 273 (19), 251 (17), 250 (100), 121 (22); HREIMS *m/z* 580.3844 (calcd for C_36_H_52_O_6_, 580.3764).

*Lapachoyl Δ8(9) junicedrate* (**7**). Compound **7** was synthesized as described for compound **1**, using diterpene **II** and quinone **IV**, yielding 124 mg (48%) of **7**: pale yellow oil; [α]^20^_D_ +72 (*c* 0.15, CHCl_3_); IR ν_max_ (film) 3378, 2932, 2864, 1772, 1728, 1692, 1684, 1461, 1294, 1170, 1071 cm^−1^; ^1^H-NMR (CDCl_3_): see Table 4; ^13^C-NMR (CDCl_3_): 37.21 (C-1), 19.52 (C-2), 37.44 (C-3), 43.78 (C-4), 53.55 (C-5), 20.73 (C-6), 34.26 (C-7), 126.82 (C-8), 139.89 (C-9), 39.76 (C-10), 25.65 (C-11), 34.20 (C-12), 31.35 (C-13), 41.03 (C-14), 170.19 (C-15), 19.72 (C-16), 19.61 (C-17), 28.65 (C-18), 183.77 (C-19), 17.97 (C-20), Quinone: 178.35, 184.50 (C-1 and C-4) 150.96 (C-2), 138.28 (C-3), 130.93, 132.09 (C-4a, C-8a), 126.69, 126.53 (C-5 and C-8), 134.74, 134.01 (C-6 and C-7), 23.65 (C-1′), 118.51 (C-2′), 133.76 (C-3′), 17.97 (C-4′), 25.73 (C-5′); EIMS *m/z* 560 [M]^+^ (3), 244 (45), 243 (14), 242 (73), 228 (16), 227 (100), 225 (14), 221 (31), 175 (17), 173 (13), 109 (11), 105 (16), 95 (10), 81 (11), 55 (14); HREIMS *m/z* 560.3135 (calcd for C_35_H_44_O_6_, 560.3138).

**Table 4:** Selected ^1^H-NMR Data of compounds **7–12**.

H	7	8	9	10	11	12
13	2.12 m	2.12 m	2.10 m	2.10 m	2.12 m	2.11 m
14 α	2.72 dd	2.72 dd	2.72 dd	2.70 dd	2.74 dd	2.72 dd
14 β	2.50 dd	2.50 dd	2.53 m	2.53 m	2.46 dd	2.45 dd
16	1.15 d (6.6)	1.15 d (6.5)	1.12 d (6.6)	1.12 d (6.6)	1.15 d (6.6)	1.14 d (6.6)
17	1.54 s	1.62 s	1.59 s	1.59 s	0.95 d (7.5)	0.93 d (7.5)
18	1.28 s	1.22 s	1.25 s	1.19 s	1.27 s	1.19 s
20	0.91 s	0.80 s	0.88 s	0.77 s	0.83 s	0.70 s
OMe	-	3.65 s	-	3.62 s	-	3.66 s
Quinone						
5 and 8	8.11 m	8.11 m	8.09 m	8.09 m	8.10 m	8.10 m
6 and 7	7.75 m	7.75 m	7.72 m	7.72 m	7.74 m	7.74 m
1’	3.29 br d	3.28 br d	2.46 m	2.46 m	3.28 br d	3.28 br d
2’	5.09 br t	5.09 br t	1.35 m	1.36 m	5.08 br t	5.09 br t
3’	-	-	1.58 m	1.58 m	-	-
4’	1.78 s	1.78 br s	0.93 d (6.6)	0.93 d (6.6)	1.78 br s	1.78 br s
5’	1.70 s	1.70 br s	0.93 d (6.6)	0.93 d (6.6)	1.70 br s	1.69 br s

*J* (Hz): 14*α*, 14*β* = 15.3; 13, 14*α* = 5.5; 13, 14*β* = 8.1.

*Lapachoyl Δ8(9) junicedrate methyl ester* (**8**). Compound **7** (60 mg, 0.107 mmol) was methylated with a solution of CH_2_N_2_ in ethyl ether, yielding 57 mg (93%) of **8**: pale yellow oil; [α]^20^_D_ +57 (*c* 0.10, CHCl_3_); IR ν_max_ (film) 2924, 2876, 1768, 1720, 1688, 1637, 1461, 1290, 1138, 1075 cm^−1^; ^1^H-NMR (CDCl_3_): see Table 4; ^13^C-NMR (CDCl_3_): 37.28 (C-1), 19.52 (C-2), 37.76 (C-3), 43.91 (C-4), 53.58 (C-5), 20.87 (C-6), 34.34 (C-7), 126.80 (C-8), 139.86 (C-9), 39.59 (C-10), 25.73 (C-11), 34.24 (C-12), 31.35 (C-13), 41.03 (C-14), 170.21 (C-15), 19.74 (C-16), 19.61 (C-17), 28.47 (C-18), 178.12 (C-19), 17.79 (C-20), 51.08 (OMe), Quinone: 178.37, 184.76 (C-1 and C-4) 150.94 (C-2), 138.24 (C-3), 130.93, 132.11 (C-4a, C-8a), 126.69, 126.53 (C-5 and C-8), 134.74, 134.01 (C-6 and C-7), 23.65 (C-1′), 118.52 (C-2′), 133.75 (C-3′), 17.97 (C-4′), 25.73 (C-5′); EIMS *m/z* 574 [M]^+^ (5), 256 (15), 245 (15), 244 (91), 243 (23), 242 (100), 235 (25), 227 (52), 175 (25), 173 (22), 121 (13), 107 (11), 105 (12), 55 (11); HREIMS *m/z* 574.3307 (calcd for C_36_H_46_O_6_, 574.3294).

*Dihydroprenyl lapachoyl Δ8(9) junicedrate* (**9**). Compound **9** was synthesized as described for compound **1**, using terpene **II** and quinone **V**, yielding 91 mg (52%) of **9**: pale yellow oil; [α]^20^_D_ +41 (*c* 0.18, CHCl_3_); IR (film) *ν*_max_ 2936, 2876, 1768, 1696, 1680, 1637, 1465, 1294, 1150, 1079 cm^−1^; ^1^H-NMR (CDCl_3_): see Table 4; ^13^C-NMR (CDCl_3_): 37.25 (C-1), 19.55 (C-2), 37.51 (C-3), 43.79 (C-4), 53.55 (C-5), 20.76 (C-6), 34.27 (C-7), 126.84 (C-8), 140.18 (C-9), 39.78 (C-10), 25.78 (C-11), 34.20 (C-12), 31.41 (C-13), 41.06 (C-14), 170.32 (C-15), 19.75 (C-16), 19.56 (C-17), 28.68 (C-18), 178.17 (C-19), 17.98 (C-20), Quinone; 183.70, 184.58 (C-1 and C-4) 151.11 (C-2), 139.02 (C-3), 130.97, 132.17 (C-4a, C-8a), 126.69, 126.57 (C-5 and C-8), 134.04, 133.80 (C-6 and C-7), 22.47 (C-1′), 37.51 (C-2′), 28.41 (C-3′), 22.36 (C-4′), 22.36 (C-5′); HREIMS *m/z*: 533.1329 [M–H–CO]^+^ (calcd for C_34_H_45_O_5_: 533.3267).

*Dihydroprenyl lapachoyl Δ8(9) junicedrate methyl ester* (**10**). Compound **9** (40 mg, 0.071 mmol), was methylated with a solution of CH_2_N_2_ in ethyl ether, yielding 36 mg (88%) of **10**: pale yellow oil; [α]^20^_D_ +39 (*c* 0.24, CHCl_3_); IR (film) *ν*_max_ 2928, 2876, 1768, 1724, 1668, 1637, 1461, 1290, 1154, 1071 cm^−1^; ^1^H-NMR (CDCl_3_): see Table 4; ^13^C-NMR (CDCl_3_): 37.30 (C-1), 19.57 (C-2), 37.51 (C-3), 43.92 (C-4), 53.59 (C-5), 20.89 (C-6), 34.36 (C-7), 126.83 (C-8), 139.08 (C-9), 39.60 (C-10), 25.77 (C-11), 34.24 (C-12), 31.41 (C-13), 41.06 (C-14), 170.31 (C-15), 19.77 (C-16), 19.62 (C-17), 28.49 (C-18), 178.15 (C-19), 17.80 (C-20), 51.14 (OMe), Quinone: 183.67, 184.50 (C-1 and C-4) 151.11 (C-2), 139.08 (C-3), 130.97, 132.17 (C-4a, C-8a), 126.69, 126.57 (C-5 and C-8), 134.03, 133.79 (C-6 and C-7), 22.46 (C-1′), 37.51 (C-2′), 28.41 (C-3′), 22.36 (C-4′), 22.36 (C-5′); HREIMS *m/z*: 577.1957 [M+H]^+^ (calcd for C_36_H_49_O_6_: 577.3529).

*Lapachoyl 17β-dihydrojunicedrate* (**11**). Compound **11** was prepared as described for compound **1**, from diterpene **III** and quinone **IV**, yielding 85 mg (42%) of **11**: brown oil; [α]^20^_D_ +20 (*c* 0.09, CHCl_3_); IR (film) *ν*_max_ 3398, 2956, 2848, 1772, 1692, 1676, 1637, 1457, 1290, 1170, 1075 cm^−1^; ^1^H-NMR (CDCl_3_): see Table 4; ^13^C-NMR (CDCl_3_): 39.83 (C-1), 19.01 (C-2), 37.96 (C-3), 43.82 (C-4), 53.06 (C-5), 23.07 (C-6), 35.00 (C-7), 29.35 (C-8), 57.50 (C-9), 39.74 (C-10), 18.85 (C-11), 35.23 (C-12), 30.88 (C-13), 40.84 (C-14), 170.32 (C-15), 20.15 (C-16), 15.00 (C-17), 29.04 (C-18), 182.00 (C-19), 14.43 (C-20), Quinone: 178.42, 184.48 (C-1 and C-4) 150.98 (C-2), 138.32 (C-3), 130.97, 132.13 (C-4a, C-8a), 126.71, 126.57 (C-5 and C-8), 133.78, 134.04 (C-6 and C-7), 23.68 (C-1′), 118.55 (C-2′), 134.78 (C-3′), 17.99 (C-4′), 25.76 (C-5′); EIMS *m/z* 562 [M]^+^ (5), 500 (20), 374 (10), 244 (43), 243 (18), 242 (100), 227 (64), 221 (16), 168 (15), 141 (16), 123 (15), 109 (18), 95 (13), 83 (12), 81 (14), 55 (19); HREIMS *m/z* 562.3280 (calcd for C_35_H_46_O_6_, 562.3294).

*Lapachoyl 17β-dihydrojunicedrate methyl ester* (**12**). Compound **11** (40 mg, 0.071 mmol) was methylated with a solution of CH_2_N_2_ in ethyl ether, yielding 37 mg (90%) of **12**: brown oil; [α]^20^_D_ +24 (*c* 0.34, CHCl_3_); IR (film) *ν*_max_ 2944, 2844, 1764, 1724, 1676, 1637, 1457, 1290, 1146, 1079 cm^−1^; ^1^H-NMR (CDCl_3_): see Table 4; ^13^C-NMR (CDCl_3_): 39.85 (C-1), 19.09 (C-2), 37.27 (C-3), 43.93 (C-4), 52.99 (C-5), 23.06 (C-6), 34.98 (C-7), 29.30 (C-8),57.52 (C-9), 38.71 (C-10), 18.89 (C-11), 35.26 (C-12), 30.87 (C-13), 40.81 (C-14), 170.26 (C-15), 20.15 (C-16), 14.95 (C-17), 28.86 (C-18), 178.07 (C-19), 14.95 (C-20), 51.11 (OMe), Quinone: 178.36, 184.41 (C-1 and C-4) 150.95 (C-2), 138.27 (C-3),130.93, 132.09 (C-4a, C-8a), 126.68, 126.53 (C-5 and C-8), 134.74, 134.00 (C-6 and C-7), 23.66 (C-1′), 118.53 (C-2′), 133.74 (C-3′), 17.98 (C-4′), 25.74 (C-5′); EIMS *m/z* 576 [M]^+^ (2), 293 (17), 244 (20), 243 (20), 242 (100), 227 (27), 123 (28), 109 (14), 95 (10), 81 (11), 55 (11); HREIMS *m/z* 576.3580 (calcd for C_36_H_48_O_6_, 576.3451).

### 3.5. HCl-EtOH-Induced Ulcer Model in *Mice [7,8,9,10,27]*

The gastroprotective activity of the compounds was assessed in the EtOH-HCl-induced gastric lesion model. Male Swiss albino mice weighing 30 ± 3 g were used. The animals were fed on certified Champion diet with free access to water under standard conditions of 12 h dark-light period, 50% relative humidity, and 22 °C room temperature. The mice were randomly distributed into groups of 7–14 animals each and fasted for 24 h with free access to water before the oral administration of test samples by gavage. The purity of the tested compounds was higher than 95% by NMR analysis. To keep the animal numbers to a minimum, dose-response studies were performed with lapachol at 5, 10 and 50 mg/kg to set the conditions for single-dose comparison of gastroprotective effect. The tested compounds, lansoprazole (20 mg/kg) or the vehicle (12% Tween 80, 10 mL/kg) were administered orally by gavage. After 50 min, all groups were orally treated with 0.2 mL of a solution containing 60% EtOH-0.3 M HCl for gastric lesion induction. Animals were sacrificed by cervical dislocation 1 h after the administration of EtOH-HCl, and the stomachs excised and inflated by injection of 5% formalin (1 mL). The ulcerated stomachs were fixed in 5% formalin for 30 min and opened along the greater curvature. The length (mm) of each lesion was measured, and the lesion index expressed as the sum of the length of all lesions. Based on previous studies on the parent diterpenes, comparison of the new compounds **1–12** was carried out at a single oral dose of 5 mg/kg. The protocols were approved by the Universidad de Talca Institutional Animal Care and Use Committee, which follows the recommendations of the Canadian Council on Animal Care. Tween 80 and lansoprazole (>98% purity by HPLC) were purchased from Sigma-Aldrich.

### 3.6. Cytotoxicity Assay *[28]*

The human cell lines MRC-5 normal lung fibroblasts (ATCC CCL-171), AGS gastric adenocarcinoma cells (ATCC CRL-1739), and Hep G2 hepatocellular carcinoma cells (ATCC HB-8065) were obtained from the American Type Culture Collection (ATCC, Manassas, VA, USA). The cells were grown as monolayers in the following media: MRC-5 and Hep G2 in minimum essential Eagle’s medium (MEM), with Earle’s salts, 2.0 mM L-glutamine (Sigma Chemical Co.) and 2.2 g/L sodium bicarbonate (Sigma Chemical Co.), supplemented with 10% heat-inactivated fetal bovine serum (FBS), 100 IU/mL penicillin, and 100 µg/mL streptomycin in a humidified incubator with 5% CO_2_ in air at 37 °C. Cell passage was maintained between 10 and 16 for MRC-5 and between 79 and 82 for Hep G2 cells. The medium was changed every 2 days. Culture media, antibiotics, and FBS were obtained from Invitrogen Corp. Human gastric adenocarcinoma cells AGS (ATCC CRL-1739) were grown as monolayers in Ham F-12 medium containing 1.0 mM L-glutamine and 1.5 g/L sodium bicarbonate, supplemented with 10% heat-inactivated FBS, 100 IU/mL penicillin, and 100 µg/mL streptomycin in a humidified incubator with 5% CO_2_ in air at 37 °C. Cell passage was maintained between 42 and 48. The medium was changed every 2 days.

Confluent cultures of the different cell lines were treated with medium containing the compounds at concentrations ranging from 1 up to 1,000 µM. The antisecretory drug lansoprazole was used as reference compound. The substances were first dissolved in DMSO and then in the corresponding culture medium supplemented with 2% FBS. The final concentration of DMSO in the test medium and controls was 1%. The cells were exposed for 24 h to test medium with or without the compound (control). Each concentration was tested in quadruplicate together with the control and repeated three times in separate experiments. At the end of the incubation the neutral red uptake assay was performed.

### 3.7. Statistical Analysis

Results were expressed as the mean ± SEM. In all experiments, statistical differences between several treatments and their respective control were determined by one-way ANOVA with Student-Newman-Keuls post-hoc test. The level of significance was set at P < 0.05.

## 4. Conclusions

The aim of this work was to synthesize diterpenylnaphthoquinones combining diterpene and quinone moieties. To determine structure-activity relationships/trends, 12 esters were prepared. The diterpenes used as building blocks comprised the diacids **I**, **II** and **III**. The naphthoquinone moieties included lapachol, dihydroprenyl lapachol, and dihydroprenyl-5,6,7,8-tetrahydrolapachol. Compounds **6**, **8** and **10** were significantly active as antiulcer agents, protecting against induced gastric lesions in mice by 74, 84 and 76%, respectively. Basal cytotoxicity of the compounds was determined on the following human cell lines: normal lung fibroblasts (MRC-5), gastric epithelial adenocarcinoma (AGS) and hepatocellular carcinoma (Hep G2). Some compounds showed selective cytotoxicity against AGS cells. Derivative **5** was the most cytotoxic product, with IC_50_ values of 69, 40 and 27 µM for fibroblasts, AGS and Hep G2 cells, respectively. Derivative **6**, with strong gastroprotective activity, was devoid of cytotoxic effect towards the selected cell lines. The new compounds had different biological effects than the building blocks and some of them were less toxic than the starting molecules.

## References

[1] WHO (2003). The World Health Report.

[2] Bahmanyar S., Ye W., Dickman P.W., Nyren O. (2007). Long-term risk of gastric cancer by subsite in operated and unoperated patients hospitalized for peptic ulcer. Am. J. Gastroenterol..

[3] Luo J., Nordenvall C., Nyren O., Adami H.O., Permert J., Ye W. (2007). The risk of pancreatic cancer in patients with gastric or duodenal ulcer disease. Int. J. Cancer.

[4] Areche C., Rodríguez J.A., Razmilic I., Yañez T., Theoduloz C., Schmeda-Hirschmann G. (2007). Gastroprotective and cytotoxic effect of semisynthetic ferruginol derivatives. J. Pharm. Pharmacol..

[5] Pertino M., Schmeda-Hirschmann G., Rodríguez J., Theoduloz C. (2007). Gastroprotective effect and cytotoxicity of terpenes from the Paraguayan crude drug “yagua rova” (*Jatropha isabelli*). J. Ethnopharmacol..

[6] Rodríguez J., Theoduloz C., Yáñez T., Becerra J., Schmeda-Hirschmann G. (2005). Gastroprotective and ulcer healing effect of ferruginol in mice and rats: Assessment of its mechanism of action using *in vitro* models. Life Sci..

[7] Rodríguez J.A., Theoduloz C., Sánchez M., Razmilic I., Schmeda-Hirschmann G. (2005). Gastroprotective and ulcer-healing effect of new solidagenone derivatives in human cell cultures. Life Sci..

[8] Schmeda-Hirschmann G., Rodríguez J.A., Astudillo L. (2002). Gastroprotective activity of the diterpene solidagenone and its derivatives on experimentally induced gastric lesions in mice. J. Ethnopharmacol..

[9] Schmeda-Hirschmann G., Astudillo L., Sepúlveda B., Rodríguez J., Theoduloz C., Yáñez T., Palenzuela J.A. (2005). Gastroprotective effect and cytotoxicity of natural and semisynthetic labdane diterpenes from *Araucaria araucana* resin. Z. Naturforsch. C.

[10] Schmeda-Hirschmann G., Astudillo L., Rodríguez J., Theoduloz C., Yáñez T. (2005). Gastroprotective effect of the Mapuche crude drug *Araucaria araucana* resin and its main constituents. J. Ethnopharmacol..

[11] Sepúlveda B., Astudillo L., Rodríguez J., Yáñez T., Theoduloz C., Schmeda-Hirschmann G. (2005). Gastroprotective and cytotoxic effect of dehydroabietic acid derivatives. Pharmacol. Res..

[12] Halle W., Spielmann H. (1992). Two procedures for the prediction of acute toxicity (LD50) from cytotoxicity data. ATLA.

[13] Schmeda Hirschmann G., Papastergiou F. (2003). Naphthoquinone derivatives and lignans from the Paraguayan crude drug "tayï pytá" (*Tabebuia heptaphylla*, Bignoniaceae). Z. Naturforsch. C.

[14] Pérez S., Estevez-Braun E., Ravelo A., Ferro A.G., Tokuda E.A., Mukainaka H., Nishino T. (2003). Inhibitory effects of lapachol derivatives on Epstein-Barr virus activation. Bioorg. Med. Chem..

[15] Barbosa T.P., Câmara C.A., Silva T.M.S., Martins R.M., Pinto A.C., Vargas M.D. (2005). New 1,2,3,4-tetrahydro-1-aza-anthraquinones and 2-aminoalkyl compounds from norlapachol with molluscicidal activity. Bioorg. Med. Chem..

[16] Silva T.M.S., Camara C.A., Barbosa T.P., Soares A.Z., da Cunha L.C., Pinto A.C., Vargas M.D. (2005). Molluscicidal activity of synthetic lapachol amino and hydrogenated derivatives. Bioorg. Med. Chem..

[17] Silva R.S.F., Costa E.M., Trindade U.L.T., Teixeira D.V., Pinto M de C. F.R., Santos G.L., Malta V.R.S., De Simone C.A., Pinto A.V., de Castro S.L. (2006). Synthesis of naphthofuranquinones with activity against *Trypanosoma cruzi*. Eur. J. Med. Chem..

[18] da Silva A.J.M., Buarque C.D., Brito F.V., Aurelian L., Macedo L.F., Malkas L.H., Hickey R.J., Lopes D.V.S., Noel F., Murakami Y.L.B. (2002). Synthesis and preliminary pharmacological evaluation of new (+/−) 1,4-naphthoquinones structurally related to lapachol. Bioorg. Med. Chem..

[19] Esteves-Souza A., Figueiredo D.V., Esteves A., Câmara C.A., Vargas M.D., Pinto A.C., Echevarría A. (2007). Cytotoxic and DNA-topoisomerase effects of lapachol amine derivatives and interactions with DNA. Braz. J. Med. Biol. Res..

[20] Muregi F.W., Ishih A. (2010). Next-generation antimalarial drugs: Hybrid molecules as a new strategy in drug design. Drug Devel. Res..

[21] Morphy R., Rankovic Z. (2005). Designed multiple ligands. An emerging drug discovery paradigm. J. Med. Chem..

[22] Morphy R., Rankovic Z. (2007). Fragments, network biology and designing multiple ligands. Drug Discov. Today.

[23] Morphy R., Rankovic Z., Wermuth C.G. (2008). Multi-target drugs: Strategies and challenges for medicinal chemists. The Practice of Medicinal Chemistry.

[24] Schmeda-Hirschmann G., Pertino M., Rodriguez J.A., Monsalve F., Droguett D., Theoduloz C. (2010). Synthesis, gastroprotective effect and cytotoxicity of new amino acid diterpene monoamides and diamides. Molecules.

[25] del Corral J.M.M., Castro M.A., Rodríguez M.L., Chamorro P., Cuevas C., San Feliciano A. (2007). New cytotoxic diterpenylnaphthohydroquinone derivatives obtained from a natural diterpenoid. Bioorg. Med. Chem..

[26] Molinari A., Oliva A., Ojeda C., Escobar J., Gallardo C., del Corral J.M., Castro M.A., Cuevas C., San Feliciano A. (2005). Synthesis, characterisation and cytotoxicity of chloro derivatives of prenylnaphthohydroquinone. Bioorg. Med. Chem..

[27] Olfert E.D., Cross B.M., McWilliam A.A. (1993). Guide to the Care and Use of Experimental Animals.

[28] Rodríguez J.A., Haun M. (1999). Cytotoxicity of *trans*-dehydrocrotonin from *Croton cajucara* on V79 cells and rat hepatocytes. Planta Med..

